# Recent Advances in Metal–Organic Frameworks and Their Derivatives for Adsorption of Radioactive Iodine

**DOI:** 10.3390/molecules29174170

**Published:** 2024-09-03

**Authors:** Li Peng, Jiali Duan, Yu Liang, Haiqi Zhang, Chongxiong Duan, Sibin Liu

**Affiliations:** 1 Department of Radiology, School of Medicine, Yangtze University, Jingzhou 434023, China; celipeng@163.com; 2 School of Materials Science and Hydrogen Engineering, Foshan University, Foshan 528231, China; luffyduan@163.com (J.D.); liangyu202407@163.com (Y.L.); zhhady@163.com (H.Z.); 3 School of Materials Science & Engineering, Sun Yat-sen University, Guangzhou 510275, China

**Keywords:** metal–organic frameworks, radioactive iodine, adsorption, mechanism

## Abstract

Radioactive iodine (^131^I) with a short half-life of ~8.02 days is one of the most commonly used nuclides in nuclear medicine. However, ^131^I easily poses a significant risk to human health and ecological environment. Therefore, there is an urgent need to develop a secure and efficient strategy to capture and store radioactive iodine. Metal–organic frameworks (MOFs) are a new generation of sorbents with outstanding physical and chemical properties, rendering them attractive candidates for the adsorption and immobilization of iodine. This review focuses on recent research advancements in mechanisms underlying iodine adsorption over MOFs and their derivatives, including van der Waals interactions, complexing interactions, and chemical precipitation. Furthermore, this review concludes by outlining the challenges and opportunities for the safe disposal of radioactive iodine from the perspective of the material design and system evaluation based on our knowledge. Thus, this paper aims to offer necessary information regarding the large-scale production of MOFs for iodine adsorption.

## 1. Introduction

Radioactive iodine (e.g., ^123^I, ^124^I, ^125^I, ^129^I, and ^131^I) is a volatile product derived from uranium fission and has the characteristics of high volatility, chemical toxicity, and radiation. However, different radioisotopes of iodine exhibit distinct characteristics (e.g., half-life) and are used in various application scenarios; for example, ^129^I has a long radioactive half-life (*t*_1/2_ = 1.57 × 10^7^ years), whereas the half-lives of ^123^I, ^124^I, ^125^I, and ^131^I are less than 60 days [1,2]. ^131^I is a radionuclide with a typical short half-life (*t*_1/2_ = 8.02 days) and has been widely used in nuclear medicine for treating thyroid disorders and as an imaging agent for disease diagnosis [3,4]. However, there are safety issues related to the storage and waste disposal of ^131^I. In particular, ^131^I easily forms aerosols when discharging off-gas due to its low boiling point and volatility; thus, it can be easily absorbed into the body through breathing or food intake if handled or disposed incorrectly [5,6]. Moreover, radioactive iodine can accumulate in the thyroid gland, whose continuous exposure to large doses of radiation causes metabolic dysfunction and cancer (e.g., thyroid cancer) [7,8,9]. Currently, wastewater discharged from hospitals is a major source of radioactive iodine used for therapeutic and diagnostic purposes [10,11]. Therefore, an appropriate and timely management of the radioactive iodine waste derived from nuclear medicine is a crucial yet challenging task.

To date, many strategies have been developed to capture radioactive iodine, including liquid scrubbing processes [12], crystallization [13], glass sintering [14], and adsorption methods [4]. In particular, the adsorption method shows great potential for the efficient capture of ^131^I owing to its simplicity, high efficiency, low energy consumption, and low maintenance cost [15,16]. The key for the adsorption method is the adsorbent [17]. However, conventional adsorbents, such as zeolites (silver-exchanged zeolites) [18,19], titanosilicates [20], macroreticular resins [21], activated carbon [22], microporous polymers [23], mesoporous silicas [24], and chalcogenide aerogels [25], suffer from limited active sites, low adsorption capacity, a poor porous structure, weak binding interaction, and lack of a long-range structural order, resulting in insufficient adsorption performance for the treatment of radioactive iodine nuclides [8,26]. Therefore, the development of high-performance adsorbents with high uptake and strong affinity for effectively capturing iodine remains a considerable challenge.

Metal–organic frameworks (MOFs) are a novel class of crystalline materials that are fabricated via the self-assembly of organic ligands with inorganic metal-containing nodes of ions or clusters [27,28]. MOFs possess fascinating characteristics, such as high surface area (up to 7000 m^2^·g^−1^), diverse structures, permanent porosity, and tunable functionality, [29] which endow them with enormous potential for the effective capture and remediation of radionuclides, [30] including uranium, [31] rhenium, [32] technetium, [33] thorium [34], and iodine [4]. To date, many MOFs and derivatives have been synthesized and used as adsorbents to capture iodine owing to their high Brunauer–Emmett–Teller (BET) surface area, exceptional porosity, abundant active site, and tunable functionality [35,36]. Compared with conventional absorbent materials (e.g., zeolites, activated carbon, and microporous polymers), MOFs and their derivatives exhibit high adsorption capacity and a fast adsorption rate, and a comprehensible mechanism has been deduced for their host–guest interactions [37,38]. Most importantly, the development of appropriate interactions between iodine and MOFs prevents the adsorbed iodine from returning to the solution [16]. Furthermore, iodine-loaded MOFs show novel properties and have potential applications in diverse fields, such as heterogeneous catalysis [36].

In recent years, although great efforts have been devoted to the design and synthesis of MOFs and their derivatives for iodine adsorption [4,8,39], to the best of our knowledge, reports on the mechanism of iodine adsorption over MOFs and their derivatives are scarce, though vastly important [39]. Therefore, we believe that a review on the application of MOFs and their derivatives as adsorbents for iodine, particularly focusing on the adsorption mechanism, will be highly desirable. In this review, we discuss the state-of-the-art mechanisms for iodine adsorption over MOFs and their derivatives, providing a brief outline of most representative examples related to the adsorption mechanism, including van der Waals interactions, complexing interactions, and chemical precipitation. Finally, the challenges and outlook for the practical application of MOFs and their derivatives are discussed. Thus, the purpose of this review is to provide a road map for the future design and development of novel MOF-based materials for iodine adsorption.

## 2. Performance and Mechanisms of Iodine Adsorption over MOFs and Their Derivatives

Although iodine has a low boiling point and is easily volatilized and sublimated, MOFs and their derivatives exhibit great potential for iodine extraction and storage, regardless of the state of iodine, i.e., whether it is in the solution or vapor phase [38,39]. In addition, iodine can be used as a structural modulator or a structure-directing agent to assist MOF synthesis [40]. The surface area, porosity, functional groups, and metal doping are crucial factors for iodine adsorption on MOFs and their derivatives. Irrespective of whether the capture is a chemical or a physical process, the primary adsorption mechanisms for iodine on MOFs and their derivatives are van der Waals interactions, complexing interactions, and chemical precipitation.

### 2.1. Van Der Waals Interactions

A high surface area and porosity play important roles in the iodine adsorption performance of MOFs. This is because a higher surface area and a larger pore volume provide more adsorption sites and more space to accommodate iodine, respectively [8]. Furthermore, the pore geometry and MOF channel shape affect the iodine adsorption performance [41]. Although a high surface area, a large pore size, and pore volume are generally beneficial for iodine adsorption over MOFs, achieving appropriate values is important to improve the adsorption performance [42]. In addition, because simple surface adsorption involves weak interactions, the adsorbed iodine molecules are often desorbed or returned to the solution [43]. Therefore, the presence of only van der Waals interactions between MOFs and iodine molecules may lead to a low adsorption rate and casual desorption.

**Figure 1:**
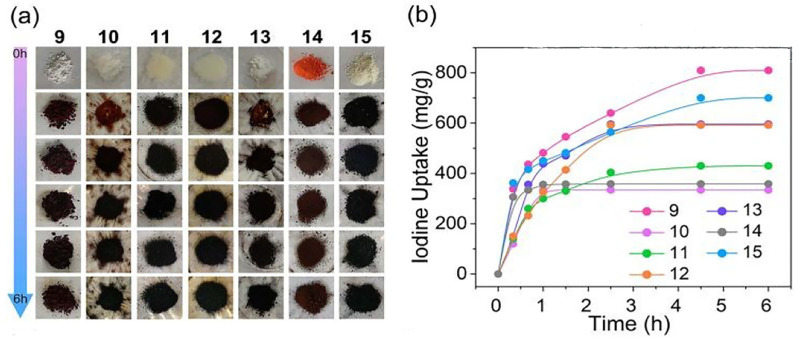
(**a**) Color changes of Th-SINAP-*n* (*n* = 9–15) MOFs after the iodine capture; (**b**) adsorption curves of iodine over Th-SINAP-*n* (*n* = 9–15) MOFs in vapor phase. Reprinted with permission from Ref. [44].

Wang et al. [44] reported the rational synthesis of high-quality Th-MOFs with tunable porosities by selecting suitable modulators and ligands. The highest void space and largest BET surface area of the Th-MOFs reached up to 74.0% and 3396.5 m^2^/g, respectively. As shown in Figure 1a, the as-synthesized Th-SINAP-*n* (*n* = 9–15) MOFs gradually became dark with time after loading iodine. Furthermore, the as-synthesized Th-SINAP-9, with the smallest pore size and BET surface area, showed the fastest sorption kinetics of less than 4.5 h and a maximum iodine uptake of 810 mg·g^−1^ (Figure 1b). This was attributed to its suitable pore opening (6.2 and 7.2 Å) compared with the kinetic diameter of iodine (4 Å), enabling iodine vapors to remain confined in small voids [45]. In addition, the as-synthesized Th-MOFs showed an excellent adsorption capacity and removal rate for iodine from cyclohexane solutions. These results indicate that the synergistic effects of the pore size, surface area, and open volume are responsible for the efficient iodine adsorption capacity over Th-MOFs.

**Figure 2:**
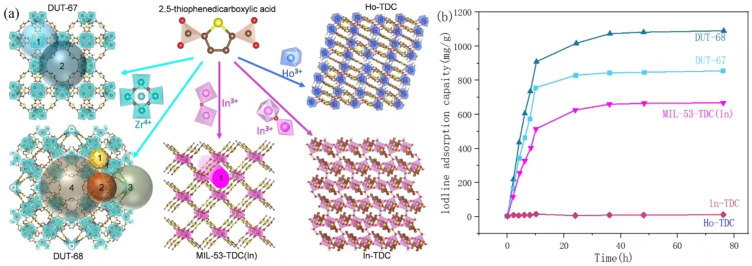
(**a**) Schematic of five thiophene-based MOFs and their pore structures; (**b**) adsorption curves of iodine over thiophene-based MOFs. Reprinted with permission from Ref. [47].

The pore structures (e.g., pore size, and pore shape) of MOFs also exert a strong influence on the iodine uptake, with a suitable matching between the pore cage and the kinetic diameter of iodine molecules favoring the capture [46]. For example, Zhang et al. [47] investigated five thiophene-based MOFs (DUT-67, DUT-68, MIL-53-TDC(In), In-DTC, and Ho-DTC) with different topologies for iodine vapor capture to systematically study the structure–property relationship between the pore structure of MOFs and their iodine adsorption performance (Figure 2a). As shown in Figure 2b, DUT-67, DUT-68, MIL-53-TDC(In), In-DTC, and Ho-DTC exhibited different iodine adsorption capacities (843, 1081, 660, 9, and 9 mg·g^−1^, respectively), which was attributed to the various pore diameter/window size ratios, and cage-type (e.g., one-dimensional (1D), two-dimensional (2D), three-dimensional (3D)) of the thiophene-based MOFs. Specifically, cage-type DUT-67 and DUT-68 provide more space for the packing of iodine molecules, whereas In-TDC and Ho-TDC exhibit small pores with only 1D channels, which hinder the loading of iodine on their outside surface.

Huang et al. [48] designed new Ni(_II_)-MOFs (SCNU-Z5) using a neoteric imidazole-tetrazole heterotopic tripodal ligand. The as-synthesized SCNU-Z5 possesses a fascinating structure with small channels running in three directions and high-connectivity pores with great potential for iodine adsorption, achieving maximum adsorption capacities for iodine in cyclohexane, water, and vapor of 442, 352, and 1680 mg·g^−1^, respectively. Mechanism studies revealed that the porosity, rather than the surface area, played a vital role in iodine adsorption. Interestingly, the partial amorphization of the SCNU-Z5 frameworks caused by the adsorbed iodine molecules improves adsorption capacity and avoids leakage without affecting the stability of materials. In addition, Sarkar et al. [49] reported new Zn-MOFs ([{Zn_3_(BTC)_2_ (H_2_O)_3_}·2.5H_2_O]*_n_*) for use as an adsorbent to capture iodine in the solution phase (water and hexane medium), showing excellent adsorption performance in water (84%) and hexane (74%) due to its large pore size (8.36 × 8.36 Å^2^) and surface area.

### 2.2. Complexing Interactions

The iodine molecule is a typical electron acceptor and, as such, is vulnerable to attack by electron-rich groups. Therefore, the rational design for improving the iodine adsorption performance of MOFs involves the introduction of electron-donor groups to frameworks, such as carbonyl groups, amines, sulfur (S) heteroatoms, π electron donors, and benzene ring molecular traps (π-π conjugate bonds) [50,51], to form strong interactions with iodine molecules through host–guest charge-transfer for their immobilization within pores [52]. However, an essential prerequisite for developing complexing interactions is the presence of unsaturated coordinate sites.

**Figure 3:**
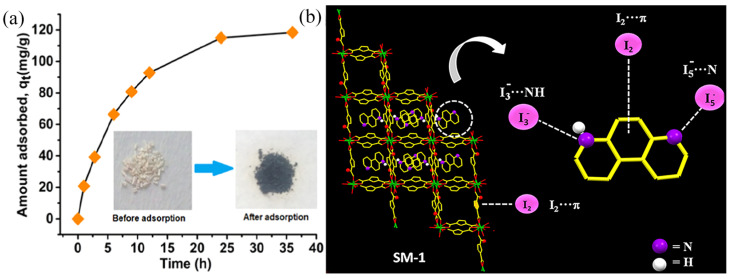
(**a**) Adsorption curves of iodine over SM–1 in cyclohexane solution, and (**b**) a plausible mechanism for iodine adsorption on SM–1. Reprinted with permission from Ref. [54].

For example, the introduction of N–rich groups into MOFs improves the iodine adsorption performance via the formation of I**⋯**N and I**⋯**π interactions. [53] Kamal et al. [54] reported amine– and imine–functionalized Mn-based MOFs ({[HPhen]_2_[Mn_3_(FDA)_4_ (H_2_O)_2_]·2H_2_O}*_n_*, denoted as SM–1) as adsorbents to capture iodine in cyclohexane solution. The resulting SM–1 frameworks contain uncoordinated N atoms and π–electron–rich moieties, resulting in an iodine uptake of 118.4 mg·g^−1^ over SM-1 and a removal efficiency of 75.4%, outperforming conventional zeolites and activated carbon. However, the adsorption capacity for iodine was lower in the vapor phase than in the liquid phase (94 mg·g^−1^ *Vs*118.4 mg·g^−1^). A plausible mechanism for iodine adsorption over SM–1 is shown in Figure 3; the free amino and imino groups and π–electron-rich moieties in the SM–1 frameworks provide abundant electrons, resulting in the formation of a charge-transfer complex with iodine. FTIR spectroscopy and PXRD results confirmed the adsorption behavior of iodine over SM–1. Subsequently, Kamal’s groups synthesized a series of functionalized MOFs for use as adsorbents of iodine in solution and vapor phases with excellent adsorption performance [55,56].

In addition, the introduction of conjugated π-electron organic ligands or electron-pair donors such as pyridine can improve the affinity between MOFs and iodine. For instance, Zhang et al. [41] reported the synthesis of novel multifunctional MOFs ([Zn_2_(tptc)(apy)_2_-*x*(H_2_O)*x*]·H_2_O) containing three phenyl rings, strongly electron-donating amino groups, and unsaturated Zn^II^ sites. The as-synthesized MOFs exhibited excellent iodine adsorption capacity (up to 216 wt.%) and removal efficiency (80.6%) in vapor and solution, which was attributed to the integrative action of the conjugated π-electron aromatic system, electron-donating amino groups, halogen bonds, and large porosity.

**Figure 4:**
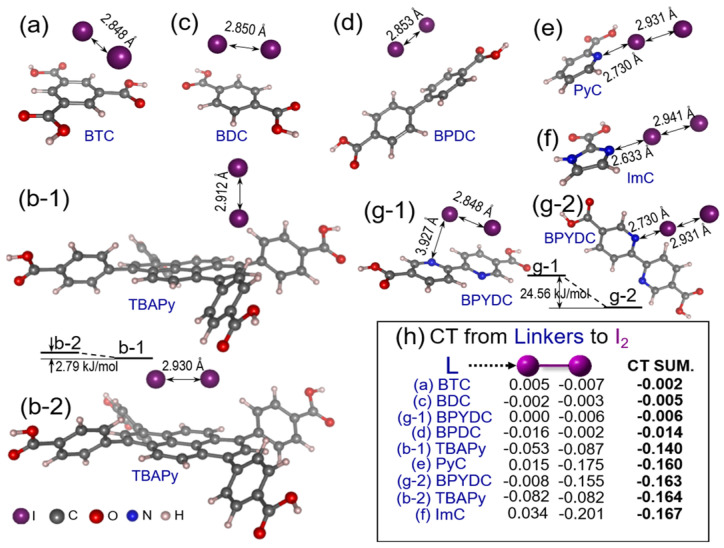
The interaction between iodine molecules and optimized geometries of various ligands: (**a**) BTC, (**b**) TBAPy, (**c**) BDC, (**d**) BPDC, (**e**) PyC, (**f**) ImC, and (**g**) BPYDC; and (**h**) their corresponding transferred charges from various ligands to iodine. Reprinted with permission from Ref. [35].

Chen et al. [35] investigated five Zr-MOFs (UiO-66, UiO-67, MOF-808, NU-1000, and MOF-867) with various carboxylate ligands for the capture of iodine under dry and humid conditions, respectively. The adsorption capacities of UiO-66, UiO-67, MOF-808, NU-1000, and MOF-867 for iodine were 660, 530, 2180, 1450, and 880 mg·g^−1^ under dry conditions, respectively. The high iodine uptake of UN-1000 was due to the presence of macrocyclic pyrene with enriched π-conjugated electron clouds. However, all Zr-MOFs showed a lower uptake in a humid environment than in a dry environment, which was attributed to the competitive adsorption of H_2_O molecules. Moreover, the incorporation of π-electron-rich components (e.g., pyridine and imidazole ligands) into MOF-808 and NU-1000 to form MOF-808-imidazole, MOF-808-pyridine, NU-1000-imidazole, and NU-1000-pyridine resulted in a decrease in the iodine adsorption capacity, which was attributed to the introduced π-electron-rich moieties blocking the pores. These results indicate that the introduction of electron-donor groups is not always conducive to iodine adsorption. In addition, the investigation of the mechanism underlying iodine adsorption over these MOFs via density functional theory (DFT) calculations revealed that the interatomic distances (I**⋯**I) in pyridine and imidazole ligands were greater than those in BTC, BDC, and BPDC ligands and that the N**⋯**I interatomic distances were smaller than the I**⋯**I distances, as shown in Figure 4. These results indicate that the N-containing and pyrene-based ligands have high affinity toward iodine.

**Figure 5:**
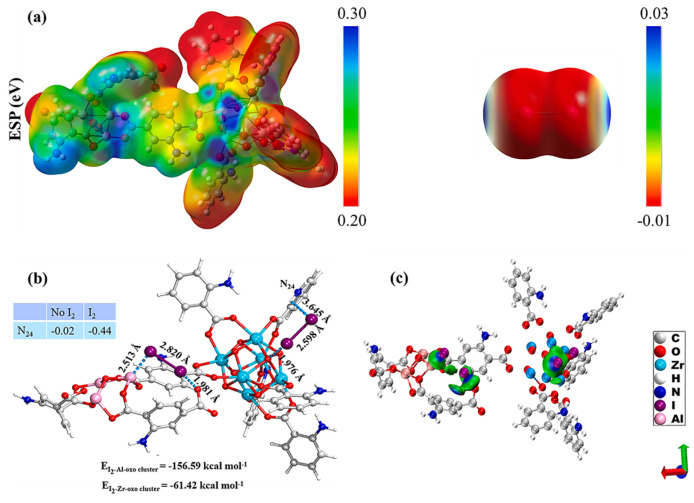
(**a**) Electrostatic potential distribution of iodine and MOF−on−MOF, (**b**) binding energies of the optimized iodine and MOF−on−MOF structure, (**c**) and intermolecular interactions between iodine and MOF−on−MOF. Reprinted with permission from Ref. [57].

Wang et al. [57] prepared amino-functionalized MOF−on−MOF composites with a core shell structure by growing NH_2_-MIL-101 MOFs on NH_2_-UiO-66 MOFs, achieving a high iodine uptake and rapid diffusion kinetics with a maximum adsorption capacity and an adsorption equilibrium time of 1930 mg·g^−1^ and 4 h, respectively. The adsorption mechanism of MOF−on−MOF for capturing iodine is shown in Figure 5. The electrostatic potential results revealed that the NH_2_-MIL-101 on NH_2_-UiO-66 frameworks displayed positive charges, opposite to the negative charges of iodine (Figure 5a). Furthermore, the Michigan charge of N atoms in -NH_2_ showed that the N atoms become more negative (from −0.02 to −0.44) (Figure 5b), indicating the formation of a strong interaction between the N atoms and iodine. The independent gradient modeling analysis in Figure 5c shows that the intermolecular interactions between the MOF−on−MOF and iodine are primarily van der Waals interactions (green area) and hydrogen bonding (blue area), suggesting the stronger affinity of the MOF−on−MOF for iodine.

**Figure 6:**
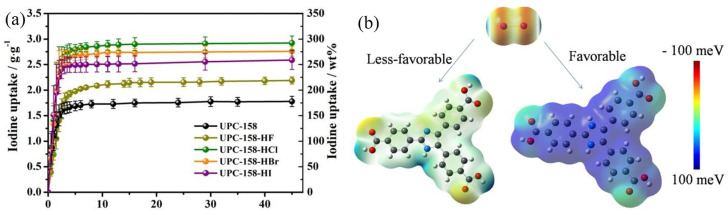
(**a**) Time−dependent iodine uptake of the as-synthesized UPC-158 and UPC-158−HX (X = F, Cl, Br, and I); and (**b**) the electrostatic potential of different states of ligand (H_3_ITTC) and iodine molecules, and their corresponding adsorption energies to the iodine molecule. Reprinted with permission from Ref. [58].

In another recent study, the impact of halogen groups on the overall iodine uptake was investigated [58]. As shown in Figure 6a, with the introduction of different halogens (e.g., F, Cl, Br, and I) into the parent UPC-158 to form UPC-158−HX (X = F, Cl, Br, and I), the iodine uptake increased up to 259% from the original value of 1770 mg/g. Notably, the iodine adsorption capacity of the UPC-158−HCl derivative was 2920 mg·g^−1^ at 343.15 K, which is the highest value recorded for MOFs. DFT calculations were used to study the difference in the adsorption ability between the neutral and acidic states. As shown in Figure 6b, although both states favored physical adsorption between the H_3_ITTC ligand and iodine molecules, the iodine uptake increased after protonation and halogenation owing to an increase in the interaction between iodine and the MOF skeleton.

**Figure 7:**
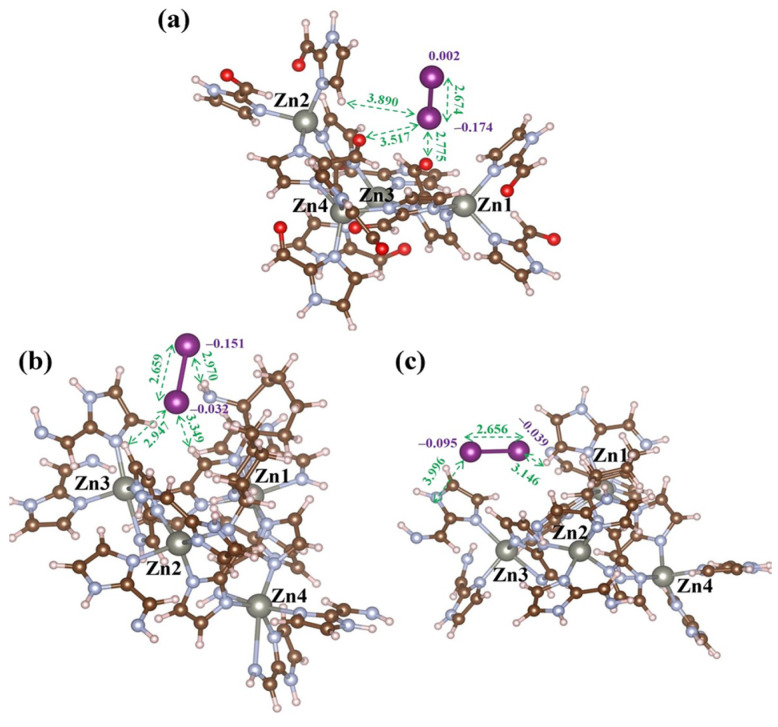
The interaction between optimized geometries of composites with an iodine molecule and the cluster of (**a**) ZIF-90, (**b**) ZIF-90-mono, and (**c**) ZIF-90-bis, respectively. Reprinted with permission from Ref. [59].

Hu et al. [59] reported the synthesis of a series of electron-rich ZIF-90 derivatives using a post-synthetic method, in which the C=N and NH_2_ groups were introduced into the ZIF-90 skeleton by converting the aldehyde groups of ZIF-90 into mono- and bis-Schiff bases. The as-synthesized ZIF-90-III derivatives with the highest amount of mono-Schiff base exhibited an iodine uptake of 6600 mg·g^−1^ from vapor and 1826 mg·g^−1^ from cyclohexane solution, which are the highest values recorded to date for MOFs. As shown in Figure 7, the distances between iodine and the imidazole-2-carboxaldehyde of ZIF-90 ranged from 2.775 to 3.890 Å, which are relatively short for the I framework compared with ZIF-90-mono (2.947–3.349 Å). However, ZIF-90-bis exhibited long distances of 3.146 and 3.996 Å between iodine and the framework. Furthermore, the I–I distances were 2.674, 2.659, and 2.656 Å in iodine-loaded ZIF-90, ZIF-90-mono, and ZIF-90-bis, respectively. These results indicate the strong interaction between ZIF-90-mono and the iodine molecule, which contrasts with the weak affinity of ZIF-90-bis toward iodine. Therefore, a higher mono-Schiff base to bis-Schiff base ratio in the MOFs can lead to stronger binding forces between iodine and the framework, thereby increasing the iodine uptake.

Owing to its high surface area, excellent thermal and chemical stability, and matching between the pore channel and the cross-section of molecular iodine, ZIF-8 shows excellent adsorption performance for iodine and great potential for iodine adsorption from an industrial perspective. For example, an iodine uptake of 125 wt.% was achieved over ZIF-8, with ~25 wt.% iodine adsorbed at the crystal surface and ~100 wt.% loaded at the cages of ZIF-8 crystals [14,60,61]. Gaining a deep knowledge of the iodine adsorption behavior is important for industrial application; however, it is difficult to differentiate between physisorption and chemisorption [62]. Fortunately, Navrotsky et al. [63] developed an aqueous solution calorimetry method to identify the adsorption behavior via measuring the binding heat of iodine for ZIF-8. The as-synthesized ZIF-8 contains an iodine/Zn ratio of 0.59 inside the cage and 0.17 on the surface. The energetics of iodine confinement per iodine in ZIF-8 were ΔH_ads_ = −41.47 ± 2.03 kJ/(mol I_2_) within the cage and ΔH_ads_ = −18.06 ± 0.62 kJ/(mol I_2_) for surface-bound iodine. The developed methodology provides theoretical support for the application of ZIF-8 to the capture of radioactive iodine and for the design of novel MOFs for capturing a wide variety of targeted molecules.

**Figure 8:**
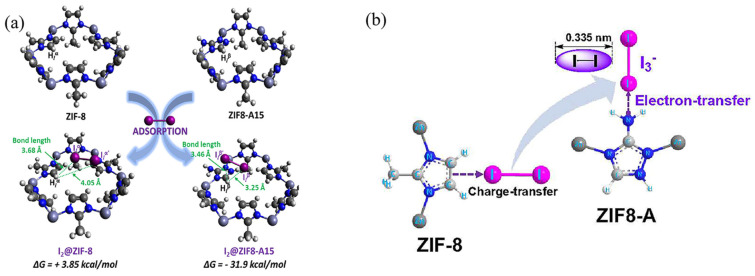
(**a**) Bond length of iodine and ZIFs (ZIF−8 and ZIF8−A) and the change of Gibbs free energy after adsorption of iodine, and (**b**) a plausible mechanism for the iodine adsorption on ZIF-8−A. Reprinted with permission from Ref. [64].

Lee et al. [64] synthesized amine-functionalized ZIF-8 (ZIF-8-A) using a post-synthetic modification method by introducing 3-amino-1,2,4-triazole (Atz) to the ZIF-8 structure. The as-synthesized ZIF-8-A exhibited superior iodine adsorption efficiency with a maximum iodine uptake of 1140 mg·g^−1^ from the solution and 3380 mg·g^−1^ from the vapor phase, outperforming pristine ZIF-8 by 870%. As shown in Figure 8a, the H**⋯**I distance in ZIF-8-A15 was shorter than that in ZIF-8 (3.25 Å vs. 4.05 Å), indicating the presence of strong interactions between iodine and the Atz ligand in ZIF-8-A15. Furthermore, the binding energy of ZIF-8-A15 (Gibbs free energy: −31.9 kcal/mol) was much lower than that of ZIF-8 (+3.85 kcal/mol), suggesting favorable conditions for iodine adsorption in ZIF-8-A15 [61]. A plausible mechanism for the iodine adsorption on ZIF-8-A is displayed in Figure 8b. The introduced amine groups (NH and NH_2_) in ZIF-8-A not only act as effective adsorption sites but also generate strong interactions with iodine molecules via electron transfer from NH and NH_2_. Moreover, the introduced amine functional groups in ZIF-8-A contribute to trap iodine molecules in the pores of the framework, preventing the adsorbed iodine from returning to the atmosphere or solution to some extent. In addition, Zhao et al. [65] reported a systematic first-principles investigation of iodine adsorption onto five different ZIFs (ZIF-Cl, SALEM-2, ZIF-8, ZIF-65, and ZIF-90) via a simulation method, providing profound insights into iodine adsorption onto ZIFs.

### 2.3. Chemical Precipitation

In fact, the unwanted release of adsorbed iodine is one of the drawbacks of the physical adsorption process due to the weak interactions between MOFs and iodine molecules. In contrast, MOFs and MOF-based composites with redox-active metal centers act as adsorbents for iodine removal with high adsorption strength via chemical adsorption through the reaction of the intrinsic redox-active metal centers with iodine, which results in precipitation [8,39]. Although the strong chemical affinity between iodine and MOFs in chemical adsorption helps avoid the adsorbed iodine molecules returning to the solution, this makes desorption a challenging task.

Yang et al. [66] reported the use of MFM-300(V^III^) with redox-active vanadium (V) centers to capture iodine. The iodine uptake of MFM-300(V^III^) reached up to 1420 mg·g^−1^, and the adsorbed iodine molecules generated helical chains inside the MOF by binding in two domains. Using electron paramagnetic resonance spectroscopy, it was demonstrated that the iodine-loaded MOFs exhibit an increase in the electrical conductivity by 700,000 times compared with the pure MFM-300(V^III^), which was attributed to the oxidized V-O(H)-V skeletons and formed iodine chains providing more transport pathways for electron transfer [67]. Furthermore, the charge transfer host–guest interactions (MOFs and iodine molecules) led to the partial oxidation of V^III^ to V^IV^ and generation of I_3_^−^. However, redox-active MOFs for iodine capture via precipitation have rarely been reported in recent years.

Apart from MOFs with intrinsic redox-active metal centers, metals (e.g., Ag, Bi, and Cu(I)) with strong chemical affinity for iodine can be loaded and highly dispersed onto the surface and pores of MOFs to capture iodine via redox reactions [68]. For example, Gong et al. [69] synthesized Ag@Cu-based MOFs by uniformly loading Ag on a HKUST-1 precursor. The effect factors, such as the Ag doping content, initial iodine anion concentration, pH, and contact time, have been investigated to evaluate their iodine capture ability. XRD and XPS analysis results indicated that Cu^0^ derived from the HKUST-1 precursor reacts with oxygen to form Cu_2_O and H_2_O_2_, while added Ag and a small amount of CuO form Ag_2_O and Cu_2_O. The generated Ag^+^ and Cu^+^ have a strong chemical affinity with iodine and produce AgI and CuI precipitates, resulting in the fast removal of iodine.

**Figure 9:**
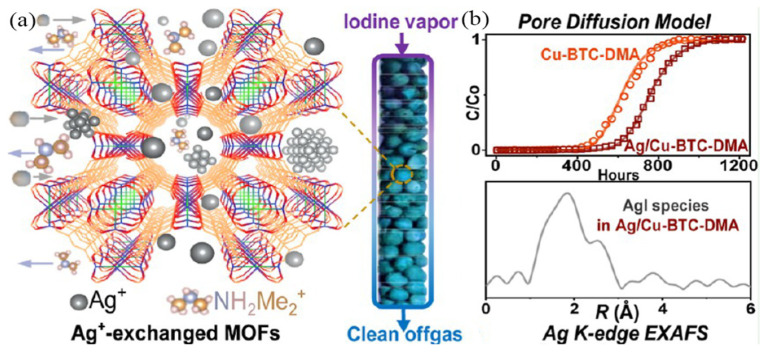
(**a**) Schematic representation of Ag/Cu-BTC-DMA, and (**b**) Ag K-edge EXAFS of iodine-loaded Ag/Cu-BTC-DMA after desorption. Reprinted with permission from Ref. [70].

Zhang et al. [70] reported the synthesis of Ag/Cu-BTC-DMA via Ag^+^ exchange with anionic MOFs. As shown in Figure 9a, Ag^+^ migrates into the interior of the Cu-BTC framework and aggregates into abundant highly dispersed Ag nanoparticles of uniform sizes (5 nm). The as-synthesized Ag/Cu-BTC-DMA MOFs showed higher iodine uptake (600 mg·g^−1^) and a much faster iodine adsorption rate than Cu-BTC-DMA (400 mg·g^−1^) without Ag. Furthermore, an EXAFS analysis indicated that the Ag species loaded within the MOFs react with iodine via chemical and irreversible adsorption to form AgI, hindering the release of the trapped iodine, as shown in Figure 9b. Meanwhile, the charge transfer from the MOF skeleton to iodine accelerates the generation of iodine ions, further promoting the formation of AgI. Furthermore, the presence of water vapor is conducive to the formation of iodine ions and their reaction with Ag^+^ species. However, when Ag^+^ was introduced into JXNU-4, ZJU-28, and ATF-1 to generate Ag-loaded MOFs, the iodine adsorption capacity was much lower than that of the pristine MOFs owing to the structure collapse of the MOFs after Ag loading. These results indicate that the incorporation of Ag into MOFs to improve the iodine uptake is not a universal strategy.

**Figure 10:**
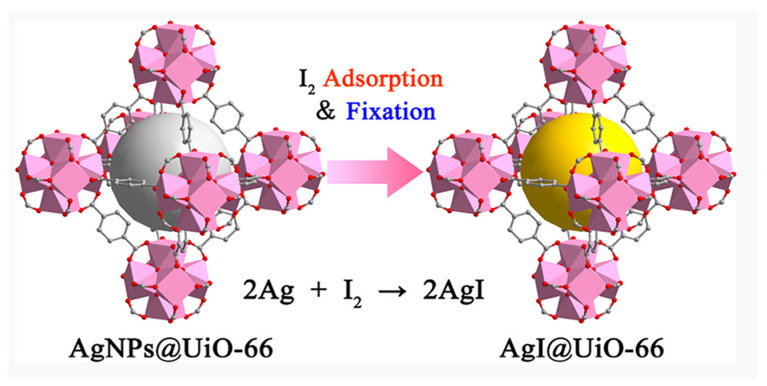
Schematic of the mechanism of iodine adsorption over AgNPs@UiO-66 MOFs. Reprinted with permission from Ref. [71].

Gu et al. [71] reported the introduction of Ag nanoparticles into UiO-66 MOFs (AgNPs@UiO-66) to improve the affinity and adsorption capacity for iodine. The as-synthesized AgNPs@UiO-66 showed high porosity and Ag-loading ability, achieving a high iodine uptake of 1260 mg·g^−1^. UV-vis spectroscopy and X-ray powder diffraction (XRD) characterizations revealed that the intrinsic pores of UiO-66 MOFs accommodate the iodine molecules, enhancing the iodine adsorption according to the mechanism shown in Figure 10. The intrinsic pores of UiO-66 MOFs provide space for accommodating iodine. Furthermore, the introduced Ag nanoparticles dispersed in the channels of UiO-66 provide adsorption sites with strong affinity to improve the interaction between iodine and the adsorbent, thereby stabilizing the absorbed-iodine molecules in the MOFs, which is beneficial for further storage processing and avoiding secondary contamination. In addition, Ag nanoparticles can be successfully doped into other MOFs (e.g., MOF-5, UiO-66-(OH)_2_, MOF-74, and Mon-POF) to synthesize Ag@MOF composites [72,73,74]. For example, the as-synthesized Ag^0^-UiO-66-(OH)_2_ MOFs showed good iodine uptake and a fast adsorption rate with an adsorption capacity of 531.98 mg·g^−1^ and a reduced adsorption equilibrium time of ~3 h [75].

In addition, Qi et al. [76] doped Cu nanoparticles onto MIL-101 to synthesize Cu/MIL-101 composites via a facile solution impregnation method. The as-synthesized Cu/MIL-101 composites exhibited better iodine uptake than the pure MIL-101 regardless of the state of iodine, i.e., whether it is in the solution or vapor state. Specifically, the maximum adsorption capacities for iodine of pure MIL-101 were 302 wt.% in vapor and 385 mg·g*^−^*^1^ in solution, respectively. However, owing to the strong chemical affinity for iodine of the doped Cu nanoparticles [8], the iodine uptake over the Cu/MIL-101 composites reached up to 342 wt.% from vapor and 432 mg·g*^−^*^1^ from solution, respectively. Moreover, the resulting Cu/MIL-101 composites showed good stability and cyclability for iodine adsorption because of the absence of the leaching of Cu nanoparticles, rendering them highly promising for practical applications.

Zhang et al. [77] reported the synthesis of two Cu-loaded MOF-303 composites (Cu^2+^-MOF-303 and Cu^0^-MOF-303) by employing MOFs as a scaffold to bind Cu^2+^ species. The as-synthesized Cu-loaded MOF-303 composites showed high structural stability during iodine trapping and excellent iodine affinity. Specifically, the iodine uptake capacities of Cu^0^-MOF-303 and Cu^2+^-MOF-303 at 353 K were 837 and 747 mg·g^−1^, respectively. Even at a high temperature of 423 K, the iodine uptake remained higher than 300 mg·g^−1^. However, DFT simulations revealed that the adsorption mechanisms for iodine over Cu^0^-MOF-303 and Cu^2+^-MOF-303 were different. Specifically, on Cu^0^-MOF-303, Cu^0^ reacts with iodine to produce CuI via a redox reaction, whereas the MOF ligands on Cu^2+^-MOF-303 chelate with Cu^2+^ and transfer charges to the trapped iodine to generate poly iodine anions, resulting in the formation of stable CuI species. These results indicate that the valence state of Cu species affect the adsorption performance for iodine.

Bismuth (Bi) has great potential for iodine removal because it can react with iodine to form compounds (e.g., BiI_3_, and BiOI) [78]. For example, Li et al. [79] developed a solvothermal method to synthesize Bi@MIL composites via mixing Bi(III) and MIL-101(Fe) MOFs. The as-synthesized Bi@MIL composites showed good adsorption capacity (200 mg·g^−1^) and rapid adsorption kinetics (only 20 min) for iodine at a neutral solution. The Bi-doped content, pH, and contact time were found to affect the adsorption performance of Bi@MIL composites for iodine. For example, when the solution pH was adjusted to 3, the iodine uptake decreased (189.6 mg·g^−1^). Although Bi@MIL composites exhibited great potential for the rapid accumulation of radioactive iodine, the adsorption capacity and cycle life need improvement.

**Figure 11:**
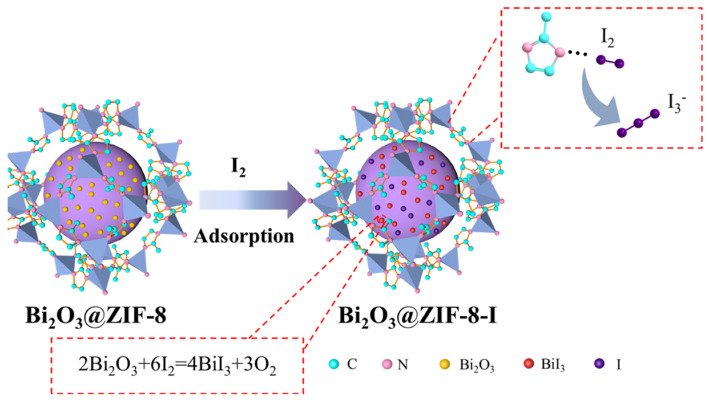
Schematic of the mechanism of iodine adsorption over the Bi_2_O_3_@ZIF−8 composites. Reprinted with permission from Ref. [80].

To address the issues of the iodine adsorption capacity of Bi-doped MOFs, Lu et al. [80] doped Bi salt (Bi(NO_3_)_3_·5H_2_O) into ZIF-8 to form Bi_2_O_3_@ZIF-8 composites using an in situ synthesis approach at room temperature. The as-synthesized Bi_2_O_3_@ZIF-8 composites exhibited excellent performance for capturing iodine in the vapor state and in solution, with maximum adsorption capacities of 298 wt.% and 751.4 mg/g, respectively, outperforming previous Bi@MIL composites. A possible mechanism for the iodine adsorption over the Bi_2_O_3_@ZIF-8 composites is shown in Figure 11, which displays three possible routes: (I) the high specific surface area and abundant porosity of the Bi_2_O_3_@ZIF-8 composites enable it to accommodate a part of iodine molecules; (II) a fraction of iodine molecules react with the N atoms of the ligand of ZIF-8 via intermolecular chemical interactions; (III) the majority of iodine molecules react with Bi to form BiI_3_ via a chemical reaction (2Bi_2_O_3_ + 6I_2_ = 4BiI_3_ + 3O_2_), forming a strong interaction that facilitates the removal of iodine. In addition, charged MOFs such as [Cu_4_I_3_(DABCO)_2_]I_3_ also exhibit excellent performance in iodine capture [81].

As a powerful tool to study the iodine adsorption performance and corresponding mechanisms of MOFs, molecular simulation can be used to design and understand MOFs for specific applications [82]. Recently, Salles et al. [42] systematically studied the influence of pores, flexibility, and cation doping of MOFs on iodine adsorption in the vapor phase via theoretical calculations. The results showed that MOFs (e.g., MIL-53, UiO-66, and MIL-101 series) containing H and NH_2_ groups exhibited excellent performance for iodine capture, with the iodine uptake increasing as follows: Cl ≈ NH_2_ ≈ H > CH_3_ > Br for the MIL-53 series, NH_2_ ≈ H > 2CH_3_ > Cl > Br > CF_3_ for the MIL-101 series, and H > NH_2_ > CH_3_ > Cl > 2CH_3_ > NO_2_ > Br for the UiO-66 series, as confirmed by experimental results [35,83]. Moreover, the simulation results evidenced that the presence of long organic moieties and large pores in MOFs endows them with great potential for iodine adsorption. This study confirmed that computational simulation is a useful tool to predict the iodine adsorption behavior of MOFs.

## 3. Conclusions and Outlook

Adsorption is a promising method for the handling or disposal of radioactive iodine to prevent health risks and long-term contamination of the environment. Compared with conventional adsorbents such as zeolites and activated carbon, MOFs are attractive candidates for iodine capture due to their fascinating physicochemical characteristics, including a high BET surface area, a tunable pore environment, abundant active sites, and ease of structural modification. Accordingly, many MOFs and their derivatives outperform conventional adsorbents in terms of the iodine uptake, which reaches even 1.0 g g^−1^ (Table 1). In this review, the main adsorption mechanisms for iodine over MOF-based materials were discussed, including van der Waals interactions, complexing interactions, and chemical precipitation. The investigation of the adsorption mechanisms is essential for elucidating the adsorption rule and conversion condition, which determine the affinity strength between MOFs and the iodine molecule. Generally, the interaction strength follows this trend: van der Waals interactions < complexing interactions < chemical precipitation.

However, despite the progress achieved in the capture of radioactive iodine using MOFs and their derivatives, there are some challenges that need to be addressed: (i) the synthesis of MOFs and their derivatives should be scaled up to the kilogram or even ton level for industrial applications. (ii) The preparation of MOFs and their derivatives usually requires harsh conditions such as high temperatures and pressures, long reaction times, and large amounts of hazardous solvents, resulting in high cost and environmental pollution. (iii) Currently, the price of MOF-based materials is very high; therefore, more attention should be paid to lower the price of raw materials and reduce energy consumption for production (e.g., operational feasibility and adaptability). (iv) Although iodine is easily captured by MOFs and their derivatives, it is often released back into the environment owing to the weak binding interaction (e.g., van der Waals interactions) between MOFs and the iodine molecule. Therefore, excellent affinity is required to ensure an efficient capture and storage rather than simple adsorption. (v) In practical applications, iodine easily reacts with volatile organic compounds (e.g., methane) to form organic iodines such as CH_3_I, which is more difficult to capture due to its ultralow concentration in the off-gas and lack of intermolecular interactions. (vi) Competitive adsorption of oxygen or solvent (e.g., H_2_O) molecules often occurs. (vii) In real operating conditions, high-quality MOFs with excellent stability against irradiation and strong affinity are required owing to the low iodine concentration and harsh adsorption conditions (i.e., high temperatures, high humidity and acidity); however, most MOFs are not sufficiently stable. (viii) The structural degradation of MOFs caused by radioactive iodine has been scarcely investigated, especially from the perspective of theoretical calculations. (ix) Some advanced techniques, such as in situ characterization and dynamic measurements, are recommended for evaluating the adsorption behavior, in particular, to determine whether iodine is adsorbed inside the channels of MOFs or on their surface. (x) The application scope of iodine-loaded MOFs with emerging properties should be broadened to fields such as catalysis and sensing. To summarize, the use of MOFs and their derivatives as adsorbents for the capture and removal of radioiodine from the aqueous and vapor phases is still immature, and more research efforts are required to improve the properties of MOFs and optimize the adsorption technology.

## Figures and Tables

**Table 1:** Summary of the reported results for the capture of iodine.

Sample	Test Temperature (°C)	Adsorption Capacity (g g^−1^)	Ref.
Th-BPYDC	75	2.23	[84]
MOF-808	80	2.18	[35]
NH_2_-MIL-101-on-NH_2_-UIO-66	80	1.93	[57]
DUT-68	80	1.08	[47]
CZ-3	75	1.15	[85]
SCNU-Z7	80	2.7	[86]
Co-IPT-IBA	75	2.88	[87]
MOF-808@PVDF	80	1.42	[88]
MIL-125	100	1.9	[89]
MIL-125_NH_2_	100	1.6	[89]
Eu-IPDA	85	1.9	[90]
Zn-ABTC	75	2.02	[91]
Co-IPT-IBA	75	2.88	[87]
{[Zn(bpaipa)]·DMF·2H_2_O}*_n_*	75	2.95	[92]
{[Zn(bpaipa)]·5H_2_O}*_n_*	75	4.51	[92]
MIL-125(Ti)_NH_2_	100	1.7	[93]
CAU-1(Al)_NH2	100	1.3	[93]
MIL-125(Ti)	100	1.4	[93]
Zn-MOF-1	75	1.25	[94]
Zn-MOF-2	75	1.96	[94]
Cu-BTC	75	1.75	[95]
ZIF-90-III	75	6.6	[59]
UPC-158-HF	70	2.19	[58]
UPC-158-HCl	70	2.92	[58]
UPC-158-HBr	70	2.75	[58]
UiO-66-FA	80	2.25	[96]
UiO-66	80	1.17	[96]
PCN-333(Al)	75	4.42	[97]
IL@PCN-333(Al)	75	7.35	[97]
UiO-66-NH-B.D	75	1.17	[98]
UiO-66-NH-T.D	75	1.33	[98]
(ZnI_2_)_3_(TPT)_2_	Room temperature	1.73	[99]

## Data Availability

Not applicable.
